# Conservative Retention of Pre‐Existing Dental Implants Contralateral to Adjuvant Radiotherapy for Maxillary Oral Squamous Cell Carcinoma: A 4‐Year Follow‐Up Case Report

**DOI:** 10.1155/crid/4881094

**Published:** 2026-06-29

**Authors:** D. Gedeonová, M. J. Fatyga, M. Hrdinka, J. Štembírek, T. Blažek, P. Hurník, J. Režnarová

**Affiliations:** ^1^ Clinic of Oral and Maxillofacial Surgery, University Hospital Ostrava, Ostrava, Czech Republic, fno.cz; ^2^ Department of Stomatology, Faculty of Medicine, University of Ostrava, Ostrava, Czech Republic, osu.cz; ^3^ Institute of Animal Physiology and Genetics, Czech Academy of Sciences, Brno, Czech Republic, cas.cz; ^4^ Clinic of Oncology, University Hospital Ostrava, Ostrava, Czech Republic, fno.cz; ^5^ Clinic of Oncology, Faculty of Medicine, University of Ostrava, Ostrava, Czech Republic, osu.cz; ^6^ Institute of Pathology and Molecular Genetics, University Hospital Ostrava, Ostrava, Czech Republic, fno.cz; ^7^ Institute of Molecular and Clinical Pathology and Medical Genetics, Faculty of Medicine, University of Ostrava, Ostrava, Czech Republic, osu.cz

**Keywords:** case report, dental implants, oral squamous cell carcinoma, osseointegration, osteoradionecrosis, peri-implantitis, radiotherapy

## Abstract

**Background:**

Dental implant survival after high‐dose head‐and‐neck radiotherapy (RT) is uncertain, and whether to explant pre‐existing implants before RT remains controversial.

**Case:**

A 58‐year‐old man with oral squamous cell carcinoma (OSCC) of the left hard palate involving the maxilla (pT4a pN2c M0) underwent left maxillectomy with bilateral neck dissection. Two right‐sided contralateral maxillary implants and three mandibular implants were retained. Adjuvant RT was delivered using IMRT/IGRT to a total dose of 70 Gy in 35 fractions to the surgical bed.

**Outcome:**

During 4 years of follow‐up with clinical examinations and panoramic radiographs, there was no clinical evidence of peri‐implantitis, implant mobility, or radiographic evidence of progressive peri‐implant bone loss around the retained implants. According to the RT treatment plan, the estimated radiation dose was 61.7 Gy at the contralateral maxillary implant sites and 29.2 Gy at the mandibular implant sites. The patient later developed an unrelated supraglottic squamous cell carcinoma treated with RT; the retained implants remained clinically stable.

**Conclusion:**

This case suggests that clinically healthy pre‐existing implants may not require routine explantation before adjuvant RT, even when implant‐site radiation exposure is substantial. Conservative retention should be accompanied by close clinical and radiographic surveillance.

**Summary**

• Pre‐existing osseointegrated implants contralateral to a maxillectomy remained stable 4 years after adjuvant 70 Gy IMRT/IGRT for maxillary oral squamous cell carcinoma, with no clinical peri‐implantitis or progressive radiographic bone loss.
• In clinically healthy implants, conservative retention with close surveillance may be reasonable even when implant‐site radiation exposure is substantial.

## 1. Introduction

Oral squamous cell carcinoma (OSCC) is the most common malignancy of the oral cavity, accounting for more than 90% of oral cancers. Treatment depends on tumor stage and location; early stage disease may be managed with single‐modality therapy, whereas advanced OSCC usually requires multimodal treatment combining surgery, radiotherapy (RT), and/or chemotherapy [1–4]. In this setting, dental assessment and long‐term oral rehabilitation are important components of multidisciplinary care.

Dental implants are widely used for functional and prosthetic rehabilitation of partially or completely edentulous patients, although implant therapy may be complicated by biological or mechanical failures [5–7]. As implant therapy becomes increasingly common, clinicians are more frequently confronted with patients who already have osseointegrated implants at the time of head‐and‐neck cancer diagnosis. Management of these pre‐existing implants before oncologic surgery and adjuvant RT remains challenging, particularly when implants are located near the planned radiation field [8–10].

RT can adversely affect both hard and soft tissues of the oral cavity. Radiation‐induced hypovascularity, hypocellularity, impaired bone turnover, and mucosal changes may contribute to peri‐implant complications, marginal bone loss, and osteoradionecrosis (ORN) [11–13]. Implant survival in irradiated patients is influenced by several factors, including radiation dose, implant location relative to the target volume, oral hygiene, systemic comorbidities, timing of implant placement, and whether implants are placed in native or grafted bone [9, 10, 14–16].

Although recent studies and systematic reviews have improved understanding of implant outcomes after head‐and‐neck RT, there is still no standardized protocol for deciding whether clinically stable pre‐existing implants should be removed before RT or retained under surveillance [14–16]. This question is particularly relevant when implants are contralateral to the surgical site but may still receive a substantial radiation dose.

This case report presents a patient with OSCC of the left hard palate involving the maxilla, in whom pre‐existing contralateral maxillary and mandibular implants were retained during surgical treatment and adjuvant RT. The case illustrates 4‐year clinical and radiographic implant stability despite substantial implant‐site radiation exposure and discusses practical implications for conservative implant retention and follow‐up.

## 2. Case Presentation

A 58‐year‐old man was referred to the Clinic of Oral and Maxillofacial Surgery, University Hospital Ostrava, for evaluation of a nonhealing palatal defect initially described by the patient as a “scratch” on the palate. Clinical examination revealed an approximately 3 × 2 cm tumor‐like lesion of the left hard palate with central ulceration and involvement of the maxillary region. The patient had no relevant systemic comorbidities, reported no regular medication or allergies, and had no previous head‐and‐neck RT. There were no diagnostic difficulties, as the lesion was clinically suspicious for malignancy. Diagnosis of OSCC was confirmed by incisional biopsy. Staging included computed tomography of the head and neck, neck ultrasonography, and panoramic radiography. The final pathological stage was pT4a pN2c M0.

The diagnostic and treatment timeline is summarized in Figure 1. Eight months before the cancer diagnosis, he had received six implants in the maxilla and three implants in the mandible, supporting fixed prosthetic restorations. At the time of diagnosis, all implants were osseointegrated and functioning (Figure 2).

**Figure 1:**
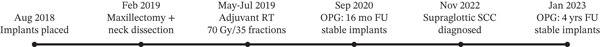
Timeline of diagnosis, surgical treatment, adjuvant radiotherapy, and follow‐up over 4 years, highlighting the continued stability of contralateral maxillary implants.

**Figure 2:**
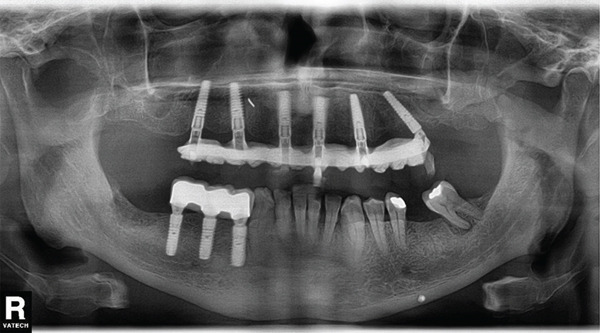
Panoramic radiograph (August 2018), 7 months before oncologic surgery, showing six maxillary and three mandibular implants supporting fixed prosthetic restorations. Four left maxillary implants were later removed during maxillectomy; two right maxillary and all mandibular implants were preserved.

In February 2019, the patient underwent left maxillectomy with bilateral neck dissection (Levels I–IV). Four implants in the left maxilla were removed as part of the oncologic resection, whereas two right‐sided contralateral maxillary implants and three mandibular implants were preserved. Primary wound healing was uneventful.

Adjuvant RT was initiated in May 2019. The patient received 70 Gy in 35 fractions to the maxillary surgical bed using IMRT/IGRT. The retained maxillary implants were contralateral to the surgical bed and were not explanted. According to the RT treatment plan, the estimated dose received at the contralateral maxillary implant sites was 61.7 Gy, whereas the mandibular implant sites received approximately 29.2 Gy. The patient completed RT without major implant‐related oral complications.

Regular follow‐up visits were scheduled, including clinical examination and panoramic radiography, approximately every 6 months. Marginal peri‐implant bone status was assessed descriptively on serial panoramic radiographs by comparing crestal bone levels around the retained implants over time. No standardized quantitative measurement protocol or CBCT‐based assessment was performed. Peri‐implantitis was defined according to the 2017 World Workshop criteria as peri‐implant inflammation with bleeding and/or suppuration on probing, increased probing depth compared with previous examinations, and progressive radiographic bone loss beyond initial crestal remodeling [17].

During 4 years of follow‐up, there were no clinical signs suggestive of peri‐implantitis, including bleeding, suppuration, increased probing depth, mucosal recession, pain, or implant mobility. No implant‐related adverse events were recorded during follow‐up. Serial panoramic radiographs did not show progressive peri‐implant bone loss around the retained implants (Figures 3 and 4).

**Figure 3:**
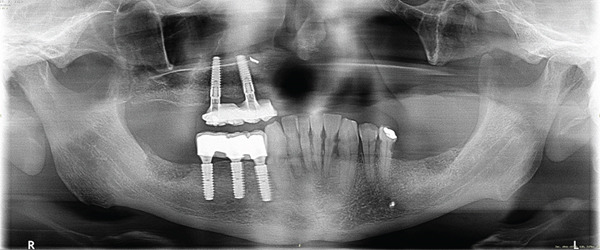
Panoramic radiograph (September 2020), approximately 16 months after completion of adjuvant radiotherapy. The retained implants show no obvious peri‐implant radiolucency or progressive crestal bone loss.

**Figure 4:**
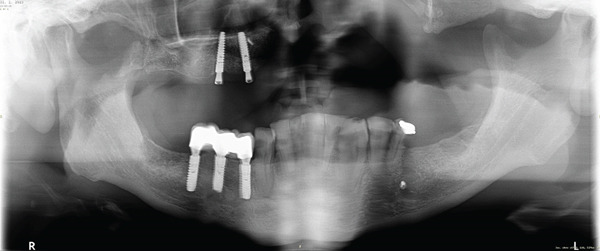
Panoramic radiograph (January 2023), 4 years after surgery and radiotherapy. Despite generalized dental deterioration related to caries and periodontitis, crestal bone around the retained implants remains stable without radiographic signs of progressive peri‐implant bone loss. The fixed prosthetic restoration supported by the two maxillary implants is absent following bridge fracture/removal; further prosthetic rehabilitation was postponed after diagnosis of a second primary tumor.

In November 2022, the patient was diagnosed with unrelated supraglottic squamous cell carcinoma (cT2 N0 M0), which was treated with definitive RT (60 Gy). The retained implants continued to show no evidence of peri‐implant disease.

In January 2023, panoramic radiography showed generalized dental deterioration related to caries and periodontitis; however, crestal bone around the retained implants remained stable (Figure 4). The absence of the upper fixed prosthetic restoration on the January 2023 panoramic radiograph reflected planned prosthetic replacement; further prosthetic rehabilitation was postponed after diagnosis of the second primary tumor. Importantly, removal of the prosthetic restoration was not related to peri‐implantitis or implant mobility. No peri‐implant treatment, soft‐tissue augmentation, vestibuloplasty, or bone augmentation was required during follow‐up.

The patient provided written informed consent for publication of clinical data and radiographic images.

## 3. Discussion

RT may compromise dental implant prognosis through its effects on bone vascularity, cellularity, remodeling capacity, and peri‐implant soft tissues [11–13]. Radiation‐induced hypovascularity, hypocellularity, impaired osteoblast function, and mucosal changes can increase susceptibility to marginal bone loss, peri‐implant inflammation, and ORN [11–13]. These mechanisms are particularly relevant when implants are located within or close to irradiated bone, where cumulative dose and local tissue response may influence long‐term stability.

Previous reports have described severe complications around implants exposed to head‐and‐neck RT. Teramoto et al. [18] reported peri‐implantitis and ORN around mandibular implants 2 years after 70 Gy RT, ultimately requiring segmental resection. Ben Slama et al. [19] similarly described ORN and implant loss after 65 Gy to the oral cavity. In contrast, larger observational studies and systematic reviews suggest that implant survival can remain acceptable in selected patients. Li et al. [9] reported a 3‐year implant survival rate of 97.4% in 58 patients who had implants before head‐and‐neck RT, whereas Neckel et al. [10] observed 97.5% survival at 3 years for implants placed after RT, with peri‐implant hard‐ and soft‐tissue changes correlating with implant‐specific radiation dose. More recently, Sukumaran et al. [20] reported outcomes in 120 patients with OSCC receiving 443 dental implants and found that implants placed before RT had a higher success rate than those placed after RT (95.1% vs. 80.1%, *p* = 0.03), whereas radiation dose category and implant site (native bone vs. fibula free flap) were not significantly associated with implant success.

Meta‐analyses also demonstrate that RT increases implant failure risk compared with nonirradiated bone: Toneatti et al. [21] reported implant survival of 91.9% in irradiated patients compared with 97% in nonirradiated patients, with an ORN incidence of approximately 3%. Schiegnitz et al. [15] reported an overall survival of 87.8% with nearly twice the risk of implant loss in irradiated bone and particularly poor outcomes when implants were placed in irradiated grafted bone. Fan et al. [14] further confirmed reduced survival in irradiated patients and emphasized increased risk in grafted bone and after radiochemotherapy. A summary of selected case reports, observational studies, and systematic reviews is provided in Table 1.

**Table 1:** Summary of selected reports on implants and radiotherapy outcomes.

Author (year)	Study type/cohort	RT dose	Implant setting	Follow‐up	Key outcome
Teramoto et al. (2016) [18]	Single case	70 Gy	Mandible, within the irradiated field	2 years	Peri‐implantitis, ORN, implant loss, segmental resection
Ben Slama et al. (2008) [19]	Single case	65 Gy oral cavity	Maxilla/mandible	40 months	ORN and implant loss
Li et al. (2022) [9]	Retrospective cohort, 58 pts, 151 implants	Median 62.4 Gy	Mixed, pre‐existing implants before RT	3 years	97.4% implant survival
Neckel et al. (2021) [10]	Observational study, 15 pts, 81 implants	Median 29–46 Gy	Maxilla/mandible, implants after RT	3 years	97.5% survival; tissue changes correlated with implant‐specific dose
Sukumaran et al. (2026) [20]	Retrospective cohort, 120 pts, 443 implants	< 60 Gy vs. > 60 Gy	Mixed, OSCC patients, pre‐RT, post‐RT, and non‐RT implants	Minimum 3 months	Higher success for implants placed before RT vs. after RT (95.1% vs. 80.1%)
Wuster et al. (2025) [22]	Retrospective cohort, 53 pts, 257 implants	Varied	Mixed, head‐and‐neck cancer patients	Mean 64 months; 5‐year analysis	Overall survival 91.8% at 5 years; adjuvant RT after FFF associated with reduced 5‐year survival (73.8%) and increased distal peri‐implant bone loss
Fan et al. (2025) [14]	Systematic review/meta‐analysis, 9 studies	Varied	Mixed, irradiated vs. nonirradiated head‐and‐neck cancer patients	Not applicable	Survival 85.6% in irradiated vs. 90.0% in nonirradiated patients; higher risk in grafted bone (RR 2.03) and with radiochemotherapy (RR 1.97)
Toneatti et al. (2021) [21]	Systematic review/meta‐analysis, 2602 implants	Varied	Mixed, irradiated vs. nonirradiated patients	Mean ~40 months	Survival 91.9% in irradiated vs. 97% in nonirradiated patients; ORN incidence ~3%
Schiegnitz et al. (2022) [15]	Systematic review/meta‐analysis, 6645 implants	Varied	Mixed, head‐and‐neck cancer patients with implants	≥ 3–5 years	Overall survival 87.8%; OR 1.97 for implant loss in irradiated vs. nonirradiated bone; highest risk in irradiated grafted bone
Prado‐Pena et al. (2024) [16]	Scoping review, 2409 implants	Varied	Mixed, pre‐existing implants undergoing RT	Not applicable	ORN observed in ~19 implants; lack of standardized guidance

The present case adds to this evidence by documenting 4‐year clinical and radiographic stability of pre‐existing implants retained during treatment for maxillary OSCC. Although the implants were anatomically contralateral to the surgical bed, treatment planning showed that the contralateral maxillary implant sites still received a substantial estimated dose of 61.7 Gy, whereas the mandibular implants received approximately 29.2 Gy. The favorable outcome may therefore not be explained simply by the absence of radiation exposure. More likely, implant stability reflected several protective factors: placement in native rather than grafted bone, preserved vascularity outside the resection site, absence of baseline peri‐implant disease, lack of implant mobility, and continued clinical/radiographic surveillance [15, 22].

Bone type is an important determinant of implant prognosis in oncological patients. Schiegnitz et al. [15] reported poorer implant survival in irradiated grafted bone than in irradiated native bone, and Wuster et al. [22] further evaluated implant survival and peri‐implant bone‐level changes after maxillary or mandibular reconstruction with free fibula flaps and found that adjuvant RT after reconstruction was associated with reduced 5‐year implant survival and increased distal peri‐implant bone loss. This distinction is relevant to the present case because the retained implants were located in native bone rather than grafted bone. Soft‐tissue conditions may also influence long‐term implant success. Studies on vestibuloplasty and optimized peri‐implant soft‐tissue management suggest potential benefits in selected irradiated patients [23, 24]. However, no peri‐implant treatment, vestibuloplasty, soft‐tissue augmentation, or bone augmentation was required in this patient during follow‐up.

There remains no standardized protocol for deciding whether clinically stable pre‐existing implants should be removed before RT or retained under surveillance. Recent reviews, including those by Prado‐Pena et al. [16] and Labidi et al. [25], highlight the need for individualized decision‐making based on implant location, radiation dose, bone type, peri‐implant status, oral hygiene, and oncological priorities. This case supports a conservative approach in carefully selected patients, but it should not be interpreted as evidence that implants exposed to high radiation doses can be retained safely in all patients.

This case report has limitations. Peri‐implant bone status was assessed descriptively on serial panoramic radiographs, and no standardized quantitative measurement protocol or CBCT‐based assessment was performed. In addition, although implant‐site dose estimates were obtained from the RT treatment plan, detailed dose–volume parameters for the peri‐implant bone were not available. Nevertheless, the 4‐year follow‐up, absence of clinical peri‐implantitis, and documented implant‐site dose provide useful information for clinical decision‐making in a setting where evidence remains limited.

## 4. Conclusion

In this case, pre‐existing implants retained during surgical treatment and adjuvant RT for maxillary OSCC remained clinically and radiographically stable for 4 years, despite substantial radiation exposure at the contralateral maxillary implant sites. Routine explantation of clinically healthy, asymptomatic implants may not be necessary in carefully selected patients; however, conservative retention should be accompanied by close clinical and radiographic surveillance.

## Author Contributions

D.G.: conceptualization, investigation, data curation, and writing—original draft; M.J.F.: investigation and writing—review and editing; M.H.: visualization and writing—review and editing; J.Š.: investigation, supervision, methodology, and writing—review and editing; T.B.: radiotherapy data curation, methodology, and writing—review and editing; P.H.: pathology data curation and writing—review and editing; J.R.: conceptualization, supervision, writing—review and editing, and project administration.

## Funding

This work was supported by the European Union under the LERCO Project Number CZ.10.03.01/00/22_003/0000003 via the Operational Programme Just Transition, by the Ministry of Health, Czech Republic (Conceptual Development of Research Organization) (RVO‐FNOs/2023), and by the Ministry of Health of the Czech Republic in cooperation with the Czech Health Research Council under Project No. NW26‐08‐00024.

## Ethics Statement

This case report was approved by the Ethics Committee of University Hospital Ostrava (Approval No. 696/2023).

## Consent

Written informed consent was obtained from the patient for publication of clinical details and radiographic images.

## Conflicts of Interest

The authors declare no conflicts of interest.

## Patient Perspective

The patient was satisfied that the retained implants remained stable during follow‐up and that no additional peri‐implant surgical intervention was required.

## Data Availability

Data sharing is not applicable to this case report because no datasets were generated or analyzed. Relevant clinical details are included in the article.
